# Comparative Formulation and Physicochemical Evaluation of Orodispersible Films Fabricated via Pneumatic and Syringe-Based 3D Printing

**DOI:** 10.1007/s11095-025-03967-4

**Published:** 2025-11-26

**Authors:** Ishwor Poudel, Nur Mita, James Scherer, Manjusha Annaji, Xuejia Kang, Oladiran Fasina, Amit K. Tiwari, R. Jayachandra Babu

**Affiliations:** 1 Department of Drug Discovery and Development, Auburn University, Auburn, AL 36830 USA; 2 Faculty of Pharmacy, Mulawarman University, Samarinda, Kalimantan Timur 75119 Indonesia; 3 Department of Biochemistry, Auburn University, Auburn, AL 36830 USA; 4 Department of Biosystems Engineering, Auburn University, Auburn, AL 36830 USA; 5 College of Pharmacy, University of Arkansas for Medical Sciences, Little Rock, AR 72205 USA

**Keywords:** 3D printing, disintegrant, gel extrusion, orodispersible film, polymer

## Abstract

**Objective:**

Orodispersible films (ODF) blend the dose accuracy of solid dosage forms and the ease of administration of liquid dosage forms, hence offer many advantages. This study investigated the feasibility of two extrusion-based 3D printing techniques (pneumatic and syringe) to fabricate ODFs in a benchtop setting.

**Methods:**

We fabricated fast-dissolving ODFs using pneumatic and syringe print heads and compared the variations in the process parameters, ease of fabrication, and characterized the properties of the final dosage forms. The variation in the printing parameters, drying time, drying temperature, and needle/nozzle types on the reproducibility and uniformity of the ODFs prepared from, these two printheads were studied. Feed materials for extrusion were selected based on rheological properties, printability, and reproducibility. An optimized ODF formulation composition was kept common and utilized for comparison.

**Results:**

The ODFs from pneumatic and syringe-based extrusion printheads consistently created bulk batches with little to no significant variation. Syringe-based extrusion showed high precision with identical dimensions, whereas pneumatic extrusion showed quick fabrication. The ODFs produced by both methods were highly reproducible and showed excellent film properties such as mechanical strength, disintegration, and dissolution. The ODFs showed adequate mechanical strength (>0.72 N/mm^2^) for packaging and transport. The disintegration time was less than a minute, and quicker dissolution within 20 min.

**Conclusion:**

Both pneumatic and syringe-based 3D printing technologies are deemed to be potentially viable alternatives for the fabrication of personalized dosage forms such as ODFs in pharmacy and clinical settings.

**Supplementary Information:**

The online version contains supplementary material available at 10.1007/s11095-025-03967-4.

## Introduction

Orodispersible films (ODFs) are attractive solid oral dosage forms that rapidly dissolve or disintegrate upon contact with saliva, making them ideal dosage forms for patients with dysphagia and those with no access to water. These dosage forms are preferred for oral formulation in geriatric and paediatric patients because of the easy swallowing of the disintegrated film. It is portable and does not require water for consumption, which makes it a patient-friendly dosage form. Despite advantages, the conventional method of manufacturing ODFs presents some technological challenges. The inconsistent film thickness and achieving uniform drug distribution across the film are difficult with large-scale manufacturing. Scaling up production from laboratory to industrial levels also introduces issues with batch consistency, equipment limitations, feed loss (for small batches), and process reproducibility [1, 2].

3D printing-based ODFs can personalize the amount of drug loaded into the film, either through an increase in the dimensions of the films or by adding multiple layers, offering the advantage of personalized pharmacotherapy based on patients’ needs and the nature of drugs and related doses [3]. Another important advantage is that they are easier to fabricate in small-scale settings such as pharmacies and hospitals for personalized care of the patients. The extemporaneous preparation of this formulation is attractive as a small batch size of ODFs could be prepared within no time, without the loss of feed materials used, and the dose and the amount of drug could be controlled perfectly to tailor for each patient [4]. There has been evidence of randomized controlled trials which concluded the acceptability, palatability, and swallowability of ODFs over other conventional formulations for neonates and infants [5]. The precision of control over the process and the acceptability by the patients make it one of the preferred fabrication techniques to address the need for personalization. The multilayered ODFs could decrease the pill burden through the concept of polypharmacy, which could be a boon for geriatric patients with dysphagia and an active multi-drug regimen. A personalized approach to manufacturing could be a great alternative to fabricate such dosage forms.

ODFs have been increasingly getting popular for 3D Printing-based manufacturing approaches because of its rapid fabrication and room for personalization of the therapy [6]. One of the reasons for 3D-printing of ODFs is the compatibility of many film-forming polymers with prevalent additive manufacturing techniques, which will be discussed further in the upcoming sections. Apart from 3D printing techniques, there are already well-established other conventional techniques for ODF fabrication, like hot melt extrusion [7], solvent casting [8], electrospinning [9]. But all these techniques do not give the flexibility to fabricate small batches intended for patient dispensing within a short period of time. Apart from that, these techniques are usually a multiple-step process and need stepwise optimization, thus making them time and resource-intensive.

Many 3D Printing-based fabrication techniques have been studied for the feasibility of ODF fabrication. Extrusion-based techniques involving microextrusion [10], fused deposition modelling (FDM) [11], hotmelt extrusion (HME) [12], solid state extrusion [13], hot melt ram extrusion [4], syringe-extrusion [14], and direct powder extrusion [15] have been utilized. Each of these techniques has its advantages and limitations. For example, FDM and HME are preferred techniques for 3D Printing fabrication based on their easy availability and translatability; however, both require extreme heat-based treatment of drugs and excipients and are not suitable for those ingredients that undergo thermal degradation. Melt ram extrusion provides an extra edge to FDM-HME, as a single step is required for printing, but thermal degradation could still be an issue. Semi-solid Extrusion (SSE) is a material extrusion technique that deposits gels, dispersions, or paste in a sequential layer on the surface, which eventually hardens upon drying to form a desired 3D-structure. SSE has been replacing the common umbrella term for all other associated technologies related to printing of liquid or semi-solid feed over the past few years, and this technology has been especially utilized in bioprinting and regenerative medicine [13]. We have explored two extrusion-based techniques in this research. Pneumatic extrusion-based techniques utilize the filtered pressurized air to extrudate the material through the selected nozzle. Syringe-pump extrusion is a mechanical extrusion that is dependent on the piston or screw driven movement, which provides more control over extrusion flow rate, achieving more precision [14].

3D-printing, though seemingly difficult to translate in a manufacturing setting, offers huge potential in extemporaneous preparation of formulations for the need-based small-scale manufacturing, in a hospital, in a compounding pharmacy, and in a clinical setting. There are still great debates on considering 3D printing more as an extemporaneous production process rather than manufacturing, as the former matches more with the promises offered by 3D Printing to deliver licensed/unlicensed medicine based on patients' needs [15]. Semisolid extrusion offers printing for specific computer-aided design (CAD) without multiple processing steps and could be a great upcoming asset for providing customized dosage forms in a hospital setting for patients through a single benchtop printer or 3D-Printing station utilizing common feedstock gel. Iria-Seoane-Viano *et al.* coined the term “nespresso style compounding” in a review in 2021 [13], which could involve a drug loading semisolid feedstock supplied to local pharmacies and compounding settings, whereby the desired drug could be mixed and dispensed to patients.

Semisolid extrusion facilitates quick dispersion if poorly water-soluble drugs are utilized, but in the case of drugs with higher drug solubility and immediate actions, it could offer a different possibility. Admixing drugs with the ready-to-print dispersion through syringe-syringe mixing, prior to printing to fabricate immediate-release dosage forms like ODFs, could be the first step to bring personalized medication into reality in the hospital pharmacy and clinic setting. There have been recent approaches of even utilizing direct powder extrusion along with solubility enhancers like cyclodextrins [17], and nanoparticular approach [18] for oral films. BCS Class I and Class III drugs could be potential targets for this technique because of their tendency to quickly solubilize in aqueous dispersions. Sumatriptan Succinate (SMT) is a BCS class III triptan drug and an effective anti-migraine medication. The water solubility of SMT is very high (101 mg/ml), which offers an excellent model drug potential to utilize in semi-solid direct extrusion processes like pneumatic extrusion and syringe-based extrusion to obtain orodispersible films.

In this research, we investigated the feasibility of various polymers for SSE-based printing and optimized a formulation to commonly explore the feasibility of two printing mechanisms in a single benchtop printer to fabricate extemporaneous batches of personalized orodispersible films for a clinical setting. Commonly used excipients for pharmaceutical manufacturing of ODFs were utilized for the study. The effect of printing techniques and parameters on the printability of such ODFs were investigated. Subsequently, an attempt to establish the relationship between feed material, surface morphology, and mechanical characterization and *in vitro* drug release was conducted.

## Materials and Methods

### Materials

Sumatriptan succinate (> 99.0%) was purchased from AA Blocks LLC (San Diego, California, USA). Polyox WSR 1105 Leo NF, Polyox WSR N10 Leo NF, was gifted by Colorcon Inc (PA, USA). Hydroxypropyl cellulose (HPC) of different grades, LM, L, SL, and SSL were gifted by Nippon Soda Co Ltd (Tokyo, Japan). Poloxamer 188 (Pluronic F68) was procured from Spectrum Chemicals (Gardena, CA, USA). Poloxamer 407 (Pluronic F127) was purchased from Sigma Aldrich (St.Louis, MO, USA). Ascorbic acid USP was purchased from Letco Medical (Decatur, AL USA). Ammonium phosphate monobasic (HPLC grade) was purchased from Fischer Scientific (Hampton, NH, USA). Glycerol was purchased from Macron Fine Chemicals (VWR International, Radnor, PA). Various grades of hydroxypropyl methyl cellulose (HPMC), low viscosity types, (Methocel E3, Methoel E5, Methocel E15) were obtained from the DOW Chemical Company (Michigan, USA), whereas Methocel E4M was gifted by Colorcon Inc (PA, USA). Polyplasdone (Crospovidone INF-10) was procured from ISF-Technologies, Inc (Jacksonville, FL, USA). Maltodextrin (Maltrin M100) was procured from the Grain Processing Corporation (Muscatine, USA). All the analytical and HPLC grade reagents required for the experiments were purchased from VWR International (Suwanee, GA, USA). An in-house MilliQ water filtration system was utilized for obtaining purified MilliQ water for the HPLC and other studies.

### Methods

The overall experimental design was divided into three sections. The first section involved extensive excipient screening and polymer screening to be appropriate for SSE. The second part involved optimization of printing dispersions for SSE, followed by the rheological characterization and testing of extrusion feasibility with both SSE-based techniques. The third part involved ODF fabrication using SSE, characterization of ODFs, and feasibility of the optimized formulation using direct extrusion and overall feasibility comparison of all three techniques regarding printing feasibility, translation, and reproducibility.

#### Excipients Screening and Polymer Selection

Several commonly utilized films forming polymers (Table S1) were investigated for their feasibility of ODF fabrication. All the formulations were initially formulated as 5%−10% of hydrogels/dispersions (glycerol as plasticizer, sodium saccharin as sweetener was common for all the formulations) and were printed using a pneumatic extrusion printhead (0.4 mm nozzle, 50 kPa- 200 kPa, printing speed (5–15 mm/s) to fabricate ODFs in small batches for comparison. The general observation for printing viability with different polymer-based printed ODF was performed using the following five qualitative characterization techniques:

- a) Printability

Printability was compared with the comparison of the shape fidelity of the printed structures. The length and breadth of the printed films (20*20*1) were measured and compared with the theoretical area of CAD design area (1 cm^2^) [19]. A shape fidelity factor > 1.1 was considered to have bad printability. The shape fidelity factor was calculated by dividing the printed cross-section area by the CAD model area. Thickness was not considered for the shape fidelity factor (SFF).

- b) Transparency

The transparency of the film was qualitatively analysed by placing an orodispersible film on a petri dish labelled with random numbers at the centre of the glass. The visibility through the film was the deciding factor for transparency.

- c) Folding Endurance and Brittleness

Three ODFs were selected at random from the printed batch and folded at least 4 to 5 times. The films that showed no sign of cracking or visible breakage were considered to have good folding endurance. The brittleness of the films was also qualitatively observed through the same experiment, and films showing visible cracks/damage within the first two folds were considered extremely brittle.

- d) Disintegration

To determine the disintegration time, point for the trial batches, the rectangular film samples were placed in a 10 mL jar with a base area of ~ 10 cm^2^. The jar was filled with 5 mL MilliQ water and shaken in a 37°C incubator at 75 rpm. The time taken for the oral film to dissolve completely was measured [20]. The experiments were conducted in triplicate.

#### Optimization of Printing Dispersions

After the initial pre-screening of polymers suitable for SSE extrusion, the best optimized formulation compositions were selected, and combinations with various excipients (plasticizers, antioxidants, sweetening agents) in different ratios were investigated. The optimized formulation composition, which was further characterized, is discussed in the results section.

- a. Rheological Characterization

The rheological properties of each dispersion system utilized for ODF preparation were tested by using a plate and cone rheometer (Malvern Bohlin CVO, Worcestershire, UK). Approximately 3–4 g of dispersions were added to the system to measure the viscosity for each formulation at both 25°C and 37°C with increasing shear rate from 0 to 100 s^−1^. All the measurements were conducted in triplicate for statistical analysis.

- b. CAD Design & 3D-Printing Parameters
  1. Pneumatic Extrusion
    Pneumatic extrusion included a printhead that could be heated to maintain the viscosity and rheological suitability of the feed, and it has a very high degree of precision with efficient response time upon extrusion, resulting in printed structures within a very short period. The pneumatic extrusion-based printhead utilized in this study was procured from Cellink (A BICO company, Gothenburg, Sweden) as a bioprinter with dynamic printheads accommodating multiple printheads for printing. A pneumatic cartridge is attached with the movable printhead assembly and connected to a filtered air compressor supply line to facilitate semi-solid extrusion, and the extrusion process is solely controlled by the pneumatic pressure. The optimized printing parameters for pneumatic extrusion, for the provided dimension, are listed in Table I.

**Table I:** Optimized Printing Parameters for Each Semi-solid Extrusion Technique

Parameters	Pneumatic (nozzle)	Pneumatic (needle)	Syringe-pump (nozzle)
ODF Dimensions (l*b*h, mm)	20*20*1	20*20*1	20*20*1
Nozzle size (mm)	0.41/0.2	0.41	0.2
Extrusion Pressure (kPa)	55/100	150	-
Printing Speed (mm/s)	6	6	6
Platform temp	45°C	45°C	45°C
Printhead temp	Ambient	Ambient	Ambient
Pre-fill	50 ms	50 ms	-
Post-fill	50 ms	50 ms	-
First Layer height	80% of nozzle size	80% of nozzle size	80% of nozzle size
Extrusion volume	-	-	1 μL/s
Retract volume	-	-	20 μL
  2. Syringe-pump Extrusion
    The syringe-pump extrusion utilizes piston-based controlled extrusion of printing dispersions from the 3 mL syringe. The syringe utilized in this technique is not exactly part of the printhead assembly; thus, it could be easily removed and refilled. The extrusion rate controls the amount of liquid deposited on the surface, which provides ultimate control, specifically with dosing and accurate prediction of the replacement cycle of the feed. The piston-driven mechanism is schematically represented in Fig. 1b. The optimized printing parameters for the syringe-pump extrusion for the given dimension are listed in Table I.

**Fig. 1:**
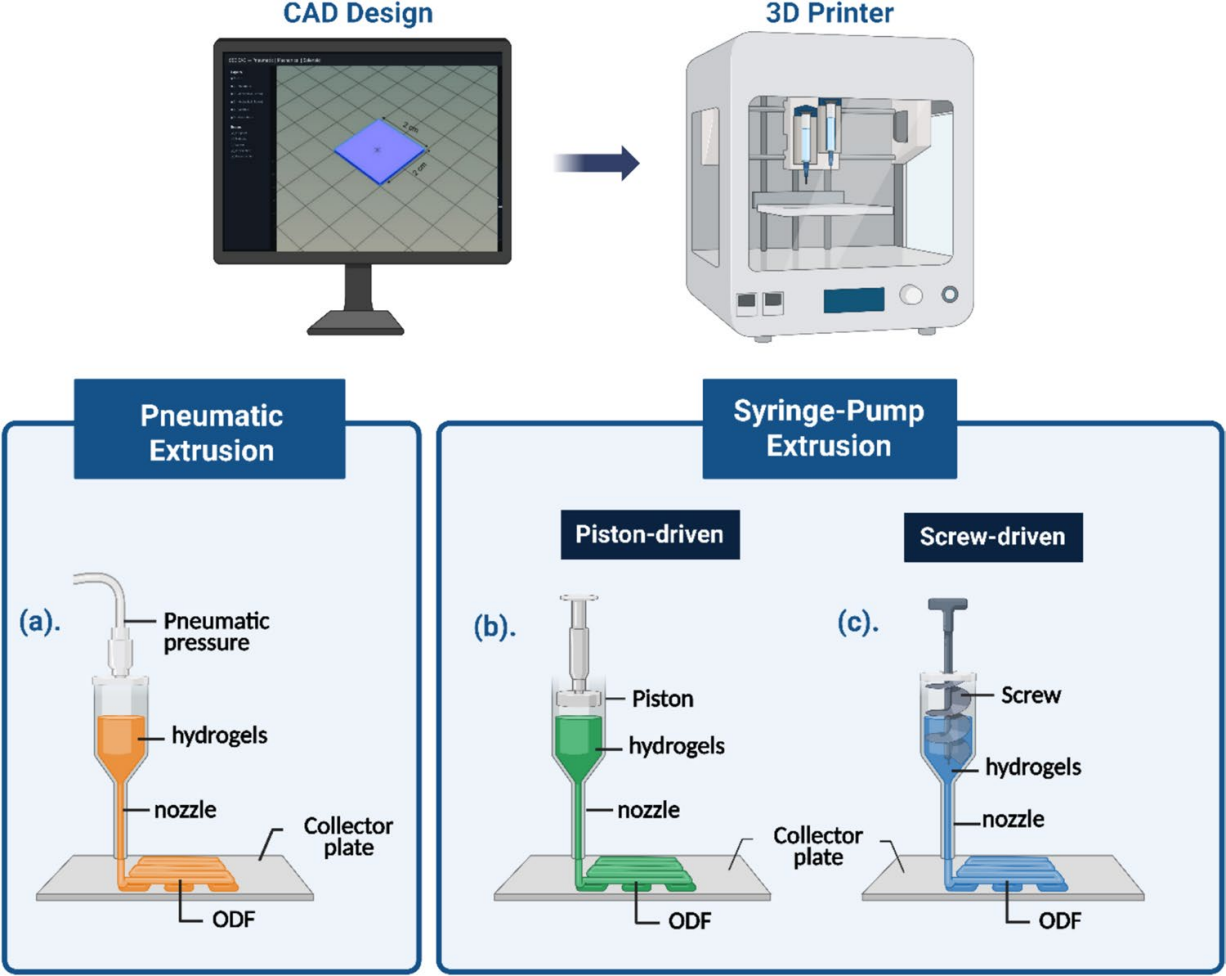
SSE techniques utilized for fabrication of ODFs and the common CAD Design: (**a**) pneumatic extrusion, (**b**) Piston-driven syringe-pump extrusion, (**c**) Screw-driven syringe-pump extrusion. Piston-driven syringe-pump extrusion was used for our study. The figure was created using www.biorender.com.

All the printed ODFs were designed using SolidWorks (SolidWorks Corp, Dassault Systèmes, Waltham, MA) and saved as a STL file (Standard Tessellation Language) before uploading into the printer. The models are visualized using Repetier-HOST software (Hot world GmbH & Co., Willich, Germany) as shown in Fig. 1. The model of the 3D-Printed ODF in the figure shows dimensions of 20 mm length, 20 mm width, and 1 mm thickness (Fig. 1). The Repetier-Host Software provides flexibility to convert the STL file into a G-code through inbuilt open-source Slic3r software (Version 1.3.1, GNU Affero Public License, version 3), and the 3D model was loaded into the bioprinter (BIOX Printer, Cellink, Gothenburg, Sweden). The printer provides a versatile printhead assembly with the ability to load three printheads at the three printhead stations. The printer also offers pneumatic pressure-assisted 3D printing through an external air compressor with a sterile filtration unit. The printing of the films was performed on a Scotchpak™ liner (1022 Scotchpak™ 3 mil, 3 M Technologies), and the printer platform was heated to 40°C to rapidly dry the film upon printing to increase the throughput. After complete fabrication of the batch, printed films were dried in a 40°C oven for a maximum of 1 h to attain completely dried films in case of pneumatic extrusion and syringe-pump extrusion. The films were stored in closely sealed tubes until further studies were performed.

#### Physicochemical Characterization of ODFs

- a. ODF Weight and Thickness

The physical appearance of the prepared films was evaluated visually, followed by measurement of the overall weight and thickness of the film. Five random films of each printed batch for each technique were measured through the length of the sides and thickness at five different locations (four corners and the center) using a micrometer screw gauge, and the mean lengths and thickness, along with the standard error of mean, were reported. The weight of the films was measured directly after the drying step, before storage in mailing tubes.

- b. Surface pH

The surface pH measurements were performed at room temperature by adding 2 mL of MilliQ water on top of unloaded and drug-loaded 4 cm^2^ films in a small glass tube. The solution is equilibrated with the pH electrode for 30–60 s until a constant reading is observed on the pH scale [21]. The readings were performed in triplicate.

- c. Thermal Characterization (Differential Scanning Calorimetry or DSC)

The thermal analysis of the blank films and the SMT-loaded films was performed using differential scanning calorimetry (DSC, Q200, TA Instruments, DE, USA). All the involved excipients, along with Sumatriptan, were weighed (< 6 mg) and placed in the aluminium pan, and an empty aluminium pan was used as a reference. The samples were equilibrated at 0°C for 10 min before ramping up to 350°C with a 10°C/min heating rate in a nitrogen atmosphere (20 psi). The obtained DSC thermograms were analysed using Universal Analysis software (TA Instruments, DE, USA).

- d. Disintegration Time

For the quantification of the disintegration time of optimized ODF batches, multiple techniques were employed. The first technique employed the rotating jar inside a shaker method, as discussed earlier. The second method involved USP disintegration apparatus in which film samples of 2 cm × 2 cm size were placed on the sample holder of disintegration apparatus and a sinker weighing 3 g was placed on the top of the sample and the test was prepared using 900 mL of distilled water maintained at 37°C. The disintegration time was noted when the film disintegrated and passed through the mesh [22]. The visibility of the disintegration was not very distinct with this technique as well. Thus, most of the disintegration times reported in this study are utilizing confirmation through both techniques and represented as mean ± SEM.

- e. Mechanical Characterization

##### Tensile Stress Test

Texture Analyzer (Stable Microsystems, Surrey, UK) with a 500 N load cell was utilized to evaluate the mechanical properties (tensile strength) of the prepared orodispersible films. The ODFs were printed at 6 cm × 1 cm × 1 cm dimensions and fixed between two clamps of the TA-DGA probe positioned at an initial distance of 2 cm. The lower clamp was locked steady while moving the upper clamp at a rate of 0.5 mm/s to pull apart the ODF until breakage occurred. The test was stopped when the film broke, and the maximum load for the force in extension was recorded. The tensile strength was calculated by dividing the tensile force by the film’s cross-sectional area. All the testing was done in triplicate.

##### Puncture Test

Texture Analyzer (Stable Microsystems, Surrey, UK) with a 500 N load cell was utilized to evaluate the puncture test on the prepared orodispersible films. The spherical probe (TA-8, 0.25-inch diameter, probe movement speed 0.5 mm/s) and cylindrical probe (TA-52, 2 mm diameter, probe movement speed 0.5 mm/s) were utilized for the puncture test at two instances separately. The force needed to puncture the film fixed in the holder (holder area = 1 cm^2^ for all the batches was measured. All the puncture testing was conducted in triplicate.

- f. Static Contact Angle Measurement

The surface wettability of the ODF film surface was estimated by the determination of static contact angle using an OCA50 goniometer with DDE/3 (Dataphysics, Filderstadt, Germany). A sessile drop technique was employed for the measurement, using water as the liquid of choice [23]. The dispense drop was 10 µL in volume, dropped at a fast speed, and visualized for the contact angle at the point of contact on the surface of the ODFs. After stabilization of the drop upon contact, the contact angle value was taken as the standard for that surface, and the contact angle was plotted against drop age to observe the change in the contact angle over time, to investigate the interaction of the solvent with the surface.

- g. Drug Content and Loading

The drug content of the printed films for each batch was determined by completely dissolving the films in a 30 mL jar with caps (lined with a Teflon liner) containing 20 mL of MilliQ water. The jars were kept at a shaker at 37°C for 2 h, vortexed for 5 min and 1 mL of supernatant was collected. The supernatant was filtered through a 0.22 µm filter and finally diluted with the mobile phase of HPLC.

- h. Surface Morphology

An orodispersible film from each batch was randomly selected after fabrication to measure the surface roughness values and to observe overall surface morphology. The surface of the ODF was observed using a 3D optical and digital microscope (Keyence VHX 6000). Keyence VHX-6000 can provide height data in accordance with the projected height to visualize the overall roughness of the surface. The profiled area usually is a planar structure with a rectangular shape focused (3 × 0.1 mm^2^) point. The standard surface roughness values, such as arithmetic mean deviation (Ra) and the maximum height of the profile (Rz) were calculated and considered as the standard roughness parameters for comparison. Six or more lines were profiled for each sample surface to reduce the statistical impact.

The microstructures and surface morphologies of the orodispersible films were observed using high-resolution FIB-SEM (ZEISS Crossbeam 550, Carl ZEISS Microscopy, Oberkochen, Germany) with an accelerating voltage of 10 kV. A thin layer of Au/Pt (~ 10 nm) was deposited on the surface, using a sputter coater, to make the surface conductive before scanning. The surface was captured at various magnifications (25 × and 100x).

- i. Dissolution Study

The in-vitro dissolution tests were conducted at pH 6.8 (USP buffer) using a USP-I basket method. The temperature was maintained at 37 ± 0.5°C, and the basket rotation was set at 75 rpm. Samples were withdrawn at predetermined time points (1, 5, 10, 15, 20, 30, 40, and 60 min). A 2 mL sample volume was collected at each time point, and a fresh dissolution medium was used to replace it to maintain sink conditions. Each sample was filtered using 0.45 µm syringe filter prior to quantification using HPLC.

#### Quantification of SMT by High Performance Liquid Chromatography (HPLC)

The concentration of SMT in the samples was quantified using an HPLC method. The HPLC instrumentation consisted of an Alliance 2695 Separation module and a 2998 photodiode array (PDA) detector. The system was interfaced to a workstation operated by Empower 3 software. The chromatographic separation was achieved using a reversed-phase C-18 column (Phenomenex, Kinetex®; 5 µm, 150 × 4.6 mm), which was set at 25°C. The mobile phase consisted of 0.05 M Monobasic ammonium phosphate: methanol at 85:15(v/v), adjusted to pH 3.3 by using orthophosphoric acid, and the method was set at a flow rate of 1 ml/min [24]. The injection volume was 20 µL. The detector was set at 227 nm. The assay samples were centrifuged for 15 min at 3000 rpm followed by filtration using 0.45 µm nylon filters to avoid potential interference due to any polymeric particulate matter. The calibration curve of SMT was plotted in triplicate in the concentration range from 2 µg/mL to 100 µg/mL.

#### Statistical Analysis

GraphPad Prism (Prism 9, GraphPad Software, San Diego, CA) was utilized for the data analysis. All the data are expressed as a mean ± SEM (Standard error of the mean) and compared using one-way Analysis of variance (ANOVA) with Tukey's Analysis to determine the level of significance as appropriate. A difference of *p* < 0.05 was considered the minimum value to establish an acceptable level of significance between samples (*n* ≥ 3). The p-value is flagged based on the level of significance (*: *P* < 0.05, **: *p* < 0.01, ***: *p* < 0.001, ****: *p* < 0.0001).

## Results

### Selection of Polymers for SSE Extrusion

The optimal CAD design utilized for the film printing (Fig. 1) was either used as it is (20 mm × 20 mm × 1 mm) or modified to 10 mm*10 mm*1 mm to reduce printing time. All the printed ODFs were dried for 30 min at 45°C and investigated for their transparency, folding endurance, brittleness, disintegration, and overall printability. Out of five parameters considered for the initial screening (Table S2), disintegration time and printability were given the highest consideration. The combination of Polyox, Poloxamer and HPC (HPC-L) was selected from the pool of polymers for the optimization of formulations and the printing parameters.

The fabricated ODFs using selected polymers follow through the 3D Printing semi-solid extrusion technology pipeline. We utilized an optimized P-F2-Z batch for this study. The viability of the process was also confirmed before moving forward with fabrication (Figure S1A). The increase in the overall weight of the deposition was reflected in the increase in the weight of the fabricated ODFs (Figure S1B). The increase in the polymeric deposition also had a relative increase in the disintegration time. The deposition of five layers (each layer using a 0.2 mm nozzle) showed increased disintegration time up to 40 s (Figure S1C). The increase in the infill of the printing also increases the weight of the final film and associated disintegration time. The increase was found to be not proportional but relative to the balance of infill and weight addition.

### Characterization of Printing Dispersions

**Fig. 2:**
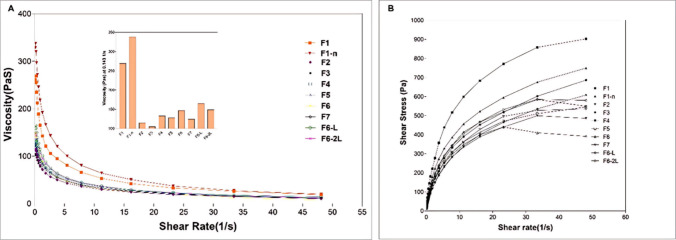
Rheological characterization of optimized formulation dispersions: (**A**) Change in the overall viscosity of the formulations with the increase in the shear rate. **B** Pseudoplastic rheograms of investigated formulations used for SSE.

The polymers selected from the pool of candidates were mixed in different ratios, and the ideal ratio for the combination is finalized using the disintegration time and multilayered viability of the obtained films. The ratio of the polymers which were selected for the dispersions preparation was optimized with the addition of glycerol (plasticizer), Sodium saccharin(sweetener), ascorbic acid (stabilizer/antioxidant), and water. The composition is listed in detail in Table II. The screened batches showed good printability and folding endurance when attempted with pneumatic nozzle-based batches. All the formulation dispersions utilized in the ODF fabrication showed a clear shear thinning property curve (flow behaviour index < 1) as the viscosity decreased with the increase in the shear rate, as shown in Fig. 2A. The viscosity range for all the dispersions was within the range for efficient extrusion (Fig. 2B). The F1 formulation containing Polyox alone was shown to have the highest viscosity (261.2 Pa · s), which increased to 328.6 Pa · s when the sample was loaded using a 0.2 mm nozzle. The shear stress when plotted against shear rate (1/s) exhibited a non-linear pseudoplastic flow behavior (Fig. 2B). The addition of poloxamer 188 has decreased the overall viscosity of the dispersion, whereas the addition of other excipients alongside HPC did not increase the apparent viscosity significantly. The drug-loaded batch, as F6-L and F7-L, seems to have appropriate viscosity for semi-solid extrusion.

**Table II:** Formulation Optimization for the Formulation Dispersions/extrusion Mix Preparation*

Batch	Polyox 1105	Poloxamer 188	HPC-L	Glycerol	SMT	Sodium Saccharin	Ascorbic acid	Water	Printability	ODF folding endurance/strength
F1	60	-	-	-	-	-	-	QS	+ + +	-
F2	60	9	-	-	-	-	-	QS	+ +	+ +
F3	60	9	-	-	-	1	-	QS	+ +	+
F4	60	9	7.5	-	-	1	-	QS	+ +	+ +
F5	60	9	-	15	-	1	-	QS	+ +	+ +
F6	60	9	7.5	15	-	1	-	QS	+ + +	+ +
F7	60	9	7.5	15	-	1	2	QS	+ +	+
F6-L	60	9	7.5	15	7.5	1	-	QS	+ +	+ +
F6-2L	60	9	7.5	15	15	1	-	QS	+ +	+ +
F7-L	60	9	7.5	15	7.5	1	2	QS	+ +	+

+ + + easiest, + + easy, + fair, -difficult, –extremely difficult

*All of this printability and ODF endurance listed for formulations in the table are for Pneumatic nozzle-based batches; QS represents Quantity Sufficient in dispersion (up to 1000 for this study)

### 3D Printing of ODFs

The optimized gel dispersions were considered for utilization in each semi-solid extrusion technology. The dispersions were intended to be used with pneumatic printhead and syringe pump extrusion. All the formulations that were printed and utilized in this study are listed for their formulation codes and composition in Table III. The bioprinter utilized in this study has a versatile printhead station with the feasibility to utilize any printhead separately or in combination. The pneumatic extrusion utilized a nozzle (Z, 22G/27G) and needles (N, 22G/27 G) for extrusion, whereas syringe pump extrusion utilized a nozzle (Z, 22G). The pneumatic extrusion process yielded 6 ODFs in 15 min for nozzle-based printing (3–4 min/ODF, using 22G nozzle), whereas around 20 min (4–5 min/ODF using 22G needle) while utilizing pneumatic needle-based printing. Syringe pump extrusion is slightly time-intensive, with fabrication time of around 24–30 min for one batch (5 min/ODF, using 22 G needle). The fabricated ODFs were allowed to dry for one hour and were stored independently in a closed container until further analysis. The ODF films containing ascorbic acid were printed in the lowest light setting of the printer and stored in a container wrapped with tin foil after fabrication. It was validated that the printing of every layer was uniform for both techniques, but showed different durations required for printing due to the difference in the printing parameters.

**Table III:** Printed Batches and Formulation Codes for all the Fabricated ODFs

Formulation Code	Formulation Composition			
	Printing Technique (Prefix)*	Optimized Dispersion	Printing nozzle/needle (Suffix)ǂ	SMT (Suffix “L”)#
P-F1-Z	P	F1	Z	-
P-F2-Z	P	F2	Z	-
P-F3-Z	P	F3	Z	-
P-F4-Z	P	F4	Z	-
P-F5-Z	P	F5	Z	-
P-F6-Z	P	F6	Z	-
P-F7-Z	P	F7	Z	-
P-F6-Z-L	P	F6	Z	L
P-F6-Z-2L	P	F6	Z	2L
P-F7-Z-L	P	F6	Z	L
P-F6-N-L	P	F7	N	L
P-F7-N-L	P	F7	N	L
S-F1-Z	S	F1	Z	-
S-F6-Z	S	F6	Z	-
S-F6-Z-L	S	F6	Z	L

*Printing technique

#L signifies drug loading (SMT); ^ǂ^ Indicates printed through either nozzle or needle- Z=Nozzle , N=Needle

### Physical Appearance, Thickness, and Weight

All the films were inspected after printing for their thickness uniformity and weight variation between batches. All the films printed under the same printing parameters did not show any significant standard deviations regarding thickness and weight, unless the printing process was compromised. The average thickness of films with 20 mm × 20 mm × 1 mm printing dimension (CAD model) ranged between 150 μm-250 μm, whereas for 20 mm × 20 mm × 0.5 mm CAD design, the thickness ranged between 75 μm and 150 μm for different batches.

### Disintegration Time

After the ODF fabrication, the disintegration time for each batch was studied. The disintegration time for the pneumatic blank batches are listed in Table IV. The disintegration time was normalized to 100 μm before comparison. The batch P-F2-Z showed the fastest disintegration time of 24 ± 2.1 s, whereas the optimized formulation with all the excipients showed the maximum disintegration time of 45.7 ± 2.1 s. For the drug-loaded batches with different technologies, the disintegration of all the final loaded films was found to be less than a minute, without any significant difference (Figure S2).

**Table IV:** Thickness and Disintegration Time of Blank Formulations

Formulation	Dimension	Thickness(μm)	Disintegration Time	Normalized DT (100 μm thickness)
P-F1-Z	20*20*0.5	52.9 ± 9.5	20 ± 3.5	37.9 ± 3.0
P-F2-Z	20*20*1	116.8 ± 4.3	28 ± 1.7	24.0 ± 2.1
P-F3-Z	20*20*0.5	31.7 ± 8.98	11.5 ± 3.5	36.1 ± 0.9
P-F4-Z	20*20*1	84.6 ± 7.3	29 ± 2	34.4 ± 3.9
P-F5-Z	20*20*0.5	57.1 ± 4.3	23 ± 1.4	40.5 ± 3.9
P-F6-Z	20*20*1	93.1 ± 14.6	42 ± 4.4	45.7 ± 7.0
P-F7-Z	20*20*1	152.4 ± 12.7	49 ± 3.7	32.4 ± 4.5

### Surface Roughness/Microscopy of Films

**Fig. 3:**
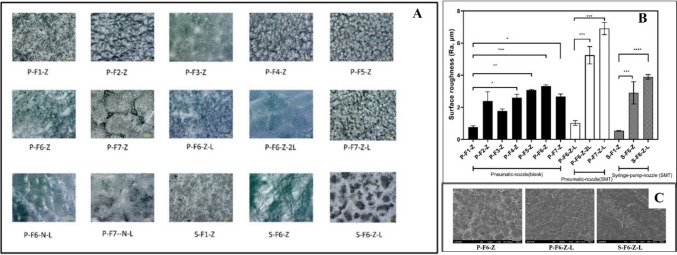
Optical microscopy of ODFs: (**A**) The high-resolution Keyence microscopic observation of surface of ODFs prepared by SSE technology. It involves pneumatic-nozzle, pneumatic needle and syringe-pump-nozzle based batches, (**B**) The surface roughness analysis of all the printed ODFs batches prepared by SSE technology, (**C**) Scanning Electron Microscopy of fabricated ODFs. The p-value is flagged based on the level of significance (*: *P* < 0.05, **: *p* < 0.01, ***: *p* < 0.001, ****: *p* < 0.0001).

The high-resolution Keyence microscopic images of all the printed ODF batchers are shown in Fig. 3A. All the images were taken under 500 × magnification. All the formulation surfaces appeared uniform with no visible damage or cracks on the surface. P-F7-G and P-F7-N-L containing ascorbic acid showed some irregularities on the surface, specifically with the remnant of air bubbles. Syringe-based extrusion batches (S-F1-Z and S-F6-Z) showed some traces of irregular structure, as the print time is long and the lower layer is exposed to the heated print bed for a longer period. To confirm if the surface is not rough microscopically, we further proceeded with the roughness analysis for all the batches.

The roughness of the printed surface was also analysed by using Keyence Microscope (VHX 6000, Keyence). The result for the surface roughness is depicted in Fig. 3B, which clearly shows that the film did not have any prominent roughness concerns. For roughness data, it was clear that F1-Z alone has the smoothest of surfaces and with the addition of excipients, has a significant increase in roughness. The SMT-loaded batch (P-F6-Z-L) did not show any roughness concerns, as expected, as the roughness (Ra) value was less than 2 μm. To confirm the absence of any irregularities in the surface, SEM analysis was performed (Fig. 3C), which clearly shows that the SSE-based ODFs are free from any structural defects. Pneumatic batch appears to have some pores on the surface, which in fact could improve the disintegration time for the formulation.

**Fig. 4:**
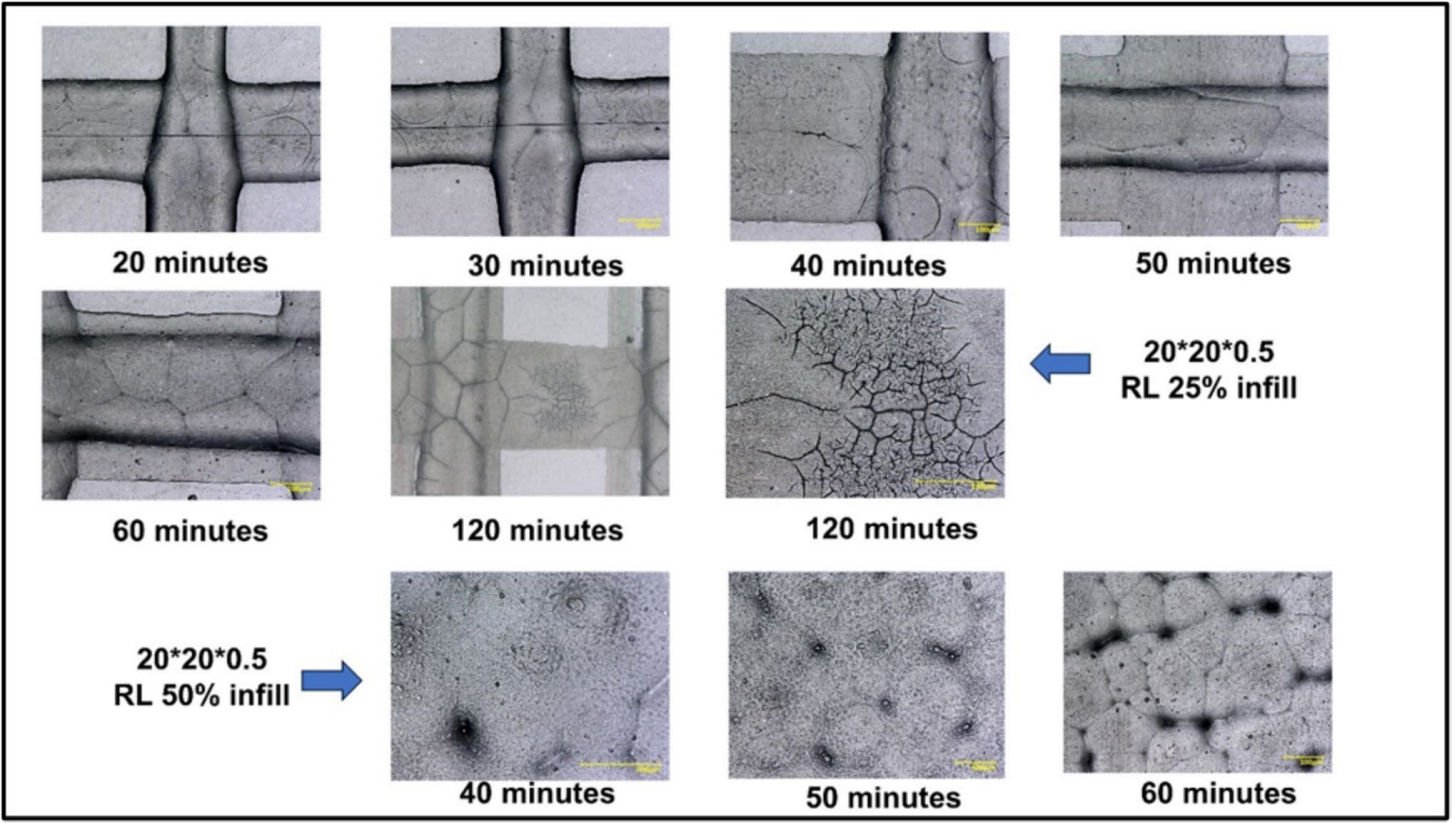
The impact of drying time on overall surface morphology of the orodispersible films (ODFs) printed by using pneumatic extrusion technology. The films prepared were of two dimensions:20*20*0.5 and 20*20*0.5. The drying time was counted from the point of fabrication to drying (both print-bed and oven drying duration).

Through our study, we have also discovered that drying time could be very crucial in modulating the disintegration of oral films. Our regular batches prepared for 50% infill are not dried for more than 1 h. The films with total drying times of more than 120 min showed development of cracks on the surface, which, upon storage for a month, resulted in brittleness. As shown in Fig. 4, the reduced infill during printing requires less time to dry, and the in-process drying should be stopped before the development of such cracks. The dried film, for less than 1 h (preferentially 30–40 min), stayed intact and remained without brittleness upon storage for up to 5 months, maintaining the drug assay within 90–110% during the storage.

### Mechanical Evaluation

#### Puncture Test

**Fig. 5:**
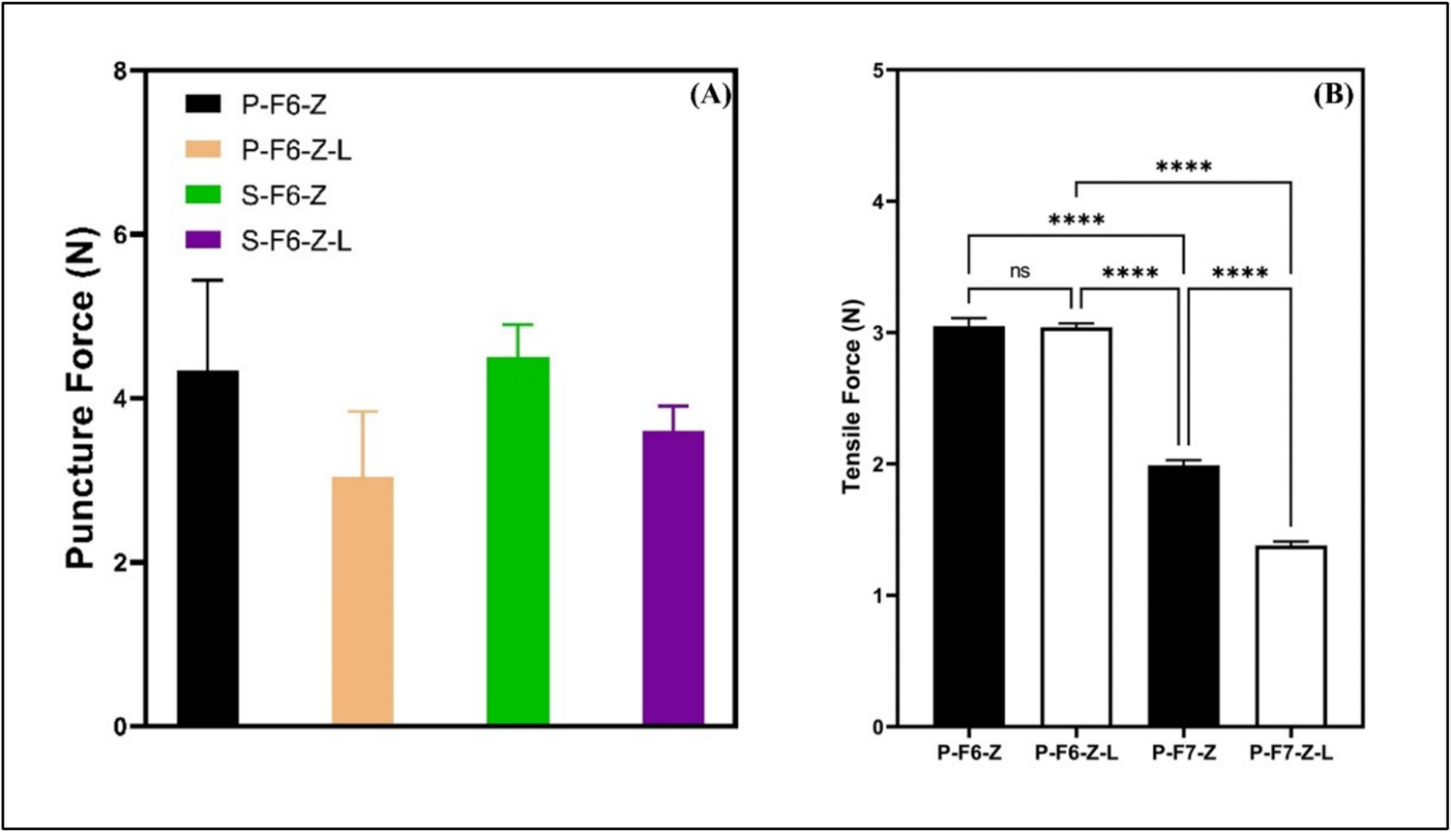
Mechanical characterization of fabricated ODFs by semisolid extrusion-based technologies: (**A**) Puncture force comparison between ODFs prepared using SSE (using TA-52 2 mm cylinder probe), (**B**) Tensile strength comparison between ODFs prepared with pneumatic extrusion. The p-value is flagged based on the level of significance (*: *P* < 0.05, **: *p* < 0.01, ***: *p* < 0.001, ****: *p* < 0.0001).

The puncture testing results for blank ODFs using pneumatic extrusion are summarized in Table V. It was performed to visualize whether any excipients have any detrimental effect on mechanical strength. A normalized puncture force was maximum for the P-F2-Z batch and lowest for P-F7-Z batch. The addition of ascorbic acid has shown a decrease in the mechanical strength. In another follow-up study, puncture test deformation of the drug-loaded ODFs was compared alongside blank films to investigate if any specific SSE technique has any impact on puncture force (Table S3, Figure S3)). The puncture force was best for ODF films fabricated with syringe-pump extrusion, with a puncture strength of 3.76 N/mm^2^, whereas one of the ascorbic acid-loaded blank films had the lowest puncture strength of 0.72 N/mm^2^ in comparison to other formulations (using 2 mm cylinder probe). There was no significant difference between batches of pneumatic and syringe-based extrusion techniques, as shown in Fig. 5A.

**Table V:** Mechanical Characterization of Blank ODFs Using Puncture Test Deformation of ODFs (using spherical probe TA-8, ¼ inch, Force normalized to 100 μm thickness)

S. code	Dimension	Puncture force (N)*	Deformation (mm)	Deformation time (s)
P-F1-Z	20*20*0.5	19.59 ± 1.9	10.4 ± 3.2	20.15 ± 6.89
P-F2-Z	20*20*1	32.39 ± 2.1	21.1 ± 2.2	41.8 ± 4.79
P-F3-Z	20*20*0.5	14.58 ± 0.7	6.62 ± 0.17	12.9 ± 0.7
P-F4-Z	20*20*1	20.96 ± 1.4	23.8 ± 3.9	47.67 ± 7.9
P-F5-Z	20*20*0.5	9.58 ± 4.6	10.03 ± 2	20.04 ± 1.2
P-F6-Z	20*20*1	11.28 ± 0.47	29.2 ± 1.23	58.32 ± 2.45
P-F7-Z	20*20*1	8.36 ± 0.38	25.9 ± 0.8	51.78 ± 1.57

#### Tensile Strength

Tensile strength studies were performed to investigate the overall strength required to break the final drug-loaded optimized batches P-F6-Z-L and P-F7-Z-L, along with their blanks. The results are summarized in Fig. 5B. The overall tensile strength in the ascorbic-loaded batch has dropped slightly lower than without ascorbic acid, but in overall strength, it does not bear high significance, which is also confirmed through the puncture test. All the specimens were strong enough that would withstand packaging.

### Surface Wettability

**Fig. 6:**
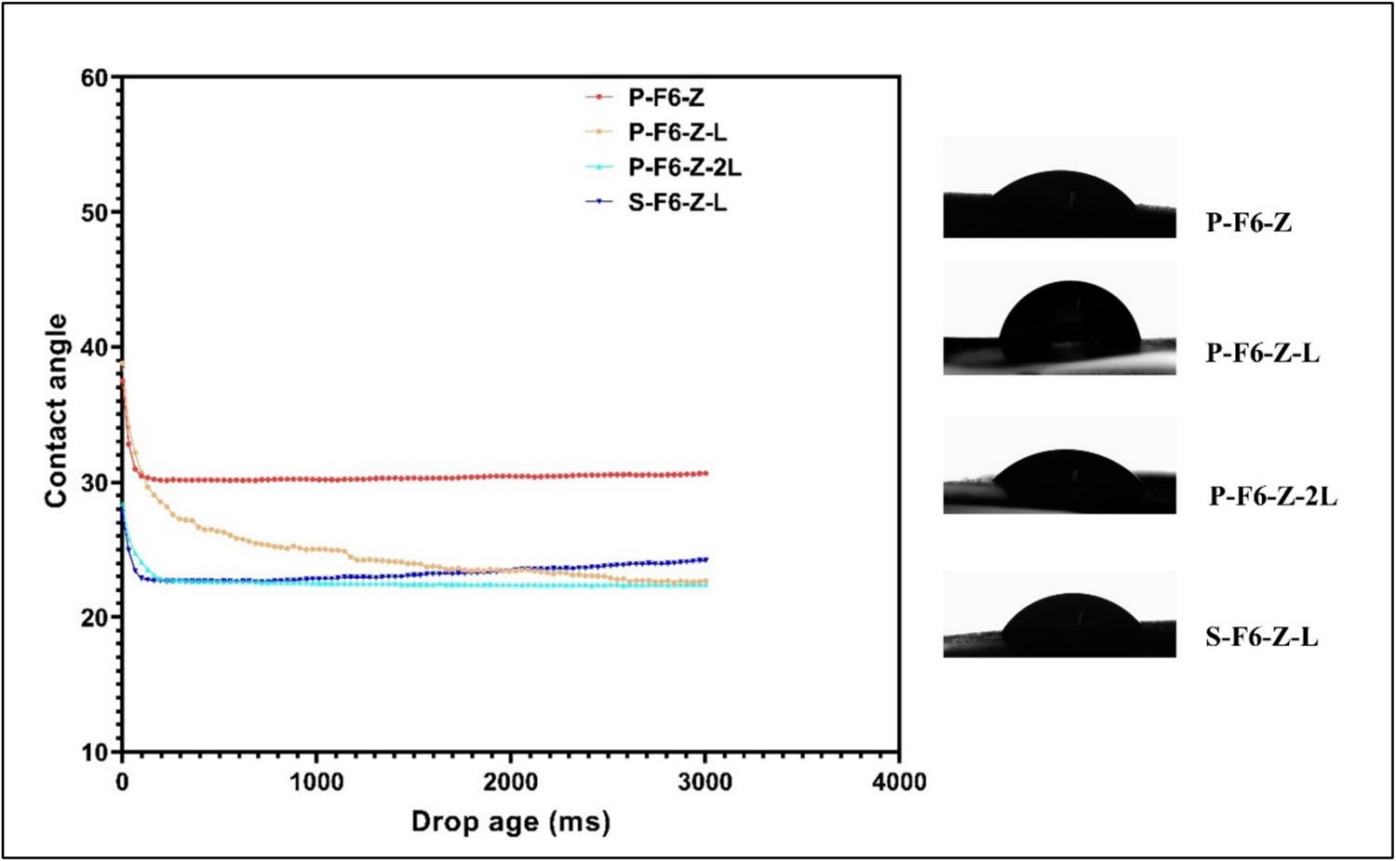
Comparison of surface wettability of the fabricated ODFs using SSE technology. All the batches were of 20*20*1 dimensions and printed using 0.4 mm nozzle.

The surface wettability data using the static contact angle experiment clearly showed that the surface of the ODF film is highly responsive to water drops, and all the experiments showed clear dissociation of the ODF surface during the experiment. The contact angle for both techniques was between 25°−30° (Fig. 6), and all surfaces showed higher affinity towards water droplets throughout the experiment (Figure S4).

### Thermal Analysis

**Fig. 7:**
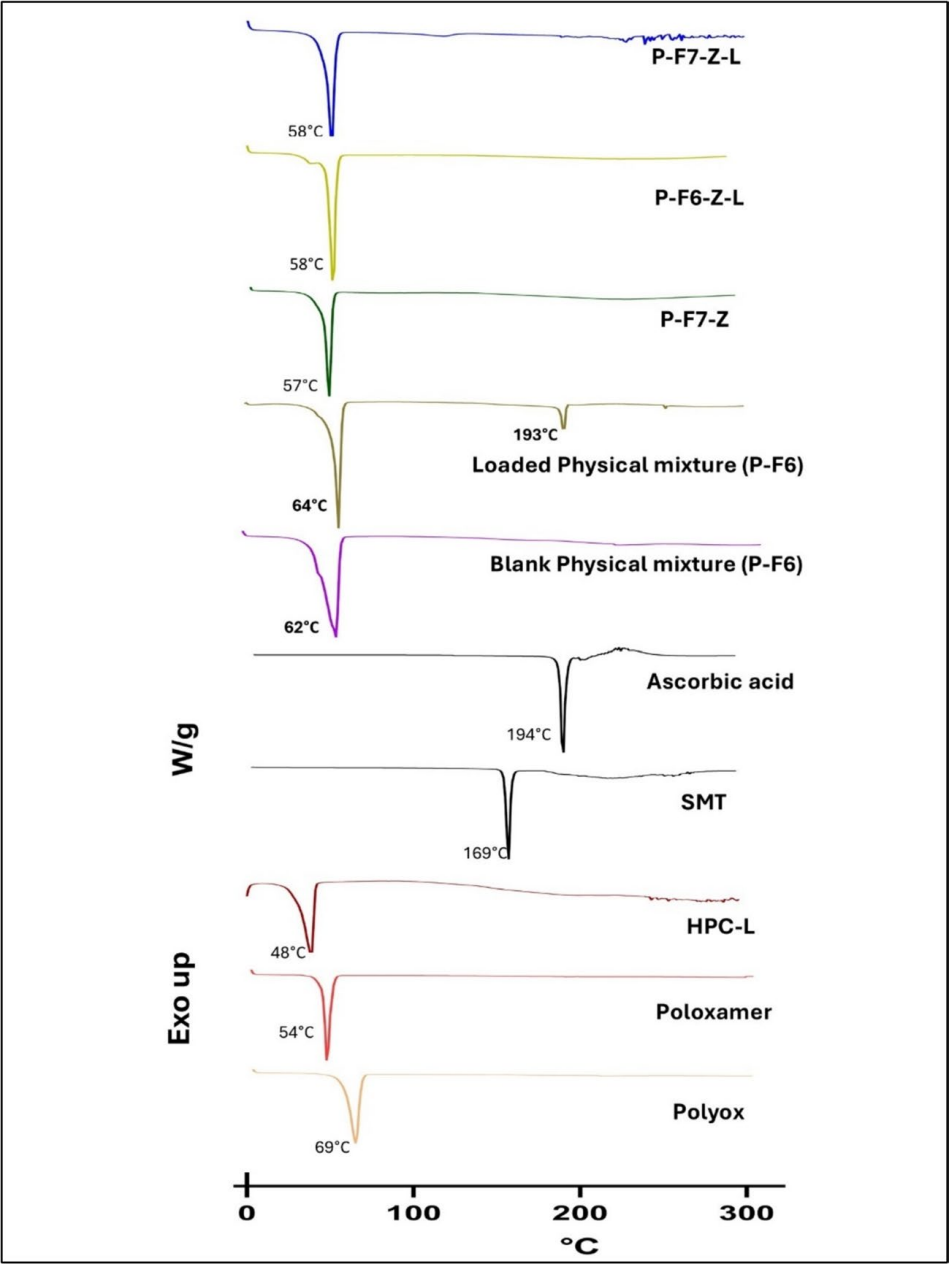
DSC comparison of orodispersible films fabricated using semi-solid extrusion, alongside physical mixtures, against pure compounds used in the dispersion-based system.

DSC analysis of the individual components of the dispersion was investigated for the thermal transition before printing of the 3D printed ODFs batch. SMT showed a sharp peak and a melting point at 169°C. For pneumatic extrusion-based batches, no melting peak of SMT was observed in drug-loaded ODF, whereas in the physical (dry) mixtures, the melting peak appeared close to drug/Ascorbic acid peak, elucidating that the drug is present in an amorphous state or solubilized state inside the ODFs. Polyox, Poloxamer, and HPC-L showed peaks at around 69°C, 54°C, and 48°C Fig. 7.

### Surface pH

The surface pH measurement is required to ensure incompatibility isn’t an impending concern *in vivo* upon administration of these ODFs. All the printed ODFs demonstrated close to neutral pH, which phases out the irritation concern in the oral mucosa upon consumption. All the formulations showed pH close to oral pH, thus making it a safe formulation to administer for oral use (Table VI).

**Table VI:** Physicochemical Properties and Assay of Drug Loaded Orodispersible Films (ODFs)

S. Code	Dimension	Weight(mg)	Thickness (μm)	DT (s)	Drug Assay (%)	pH
P-F6-Z-L	20*20*1	130.4 ± 7.2	322.1 ± 10.9	55.3 ± 5	102.3 ± 8.2	6.76 ± 0.04
P-F7-Z-L	20*20*1	95.2 ± 7.5	198.5 ± 15.1	44 ± 5	92.6 ± 5.5	6.64 ± 0.06
P-F6-N-L	20*20*0.5	59.6 ± 6.4	150.8 ± 12.6	27 ± 4	105.6 ± 3.5	6.8 ± 0.03
P-F7-N-L	20*20*0.5	72.9 ± 3.8	173.3 ± 7.9	33.3 ± 0.7	105.8 ± 10	6.74 ± 0.02
S-F6-Z-L	20*20*1	83.03 ± 1.8	186 ± 7.6	65 ± 8	102.8 ± 6.48	6.83 ± 0.01

### Drug Loading and Assay

The drug loading and assay of all the loaded batches were investigated. The theoretical concentrations anticipated from the optimized film for pneumatic printed batch is 10–25 mg/ODF and the achieved drug concentrations were within the range of 95–105% of the theoretical concentrations. We focused on a smaller concentration (~ 10 mg) during the optimization; however, it could be translatable for higher doses as well, provided that palatability concerns should be considered. The drug content of ODFs was quantified using an HPLC method. The calibration curve for SMT from 2 µg/mL to 100 µg/mL showed linearity (R^2^ = 0.999). All the drug-loaded batches showed consistent drug loading and uniformity. The overall physicochemical properties of the drug-loaded batch are discussed in Table VI. All the drug-loaded batches showed dimensional and weight uniformity between samples printed within each printing technology and exhibited acceptable disintegration time and content uniformity. The drug loaded batches were kept in closed containers in a dark place at room temperature for 3–5 months and the assay of the batches were achieved within limits of 90–110%.

### ODF Release Studies

The dissolution pattern of the orodisperible films clearly showed rapid dissolution (> 50% within first 1–5 min) of the tested final batches. We investigated the impact of infill, showing smaller infill ratio facilitates the dissolution rate even faster as shown in Figure S5(A). The impact of loading didn’t have significant effect in the dissolution rate as shown in Figure S5(B). The films obtained from either of the SSE techniques demonstrated similar dissolution profile as shown in S5(C). The ODFs with ascorbic acids resulted in relatively rapid burst release, as evident from Figure S5(D).

## Discussion

Orodispersible films formed through semi-solid extrusion was found to be very effective and efficient in terms of printability, shape fidelity and drug loading. To attain something as ambitious as getting a personalized dosage form in clinics, the practicability of this technique and its compatibility with the commonly utilized polymers in conventional formulations must be studied. The clinical practice could be feasible even in veterinary settings to regulate dosage between animals of different body weights. There have been some studies that discussed the utilization of ODFs for extemporaneous dosage forms for animal clinic settings to deliver different dosages of prednisolone to animals [16]. It is a good starting point for seeing the immediate impact of 3D printing-based medicines in the clinical environment, but the throughput of preparation time for such batches will always be a challenge. In this study, the selection of multiple polymers in the formulation was anticipated to bring in processing issues, which was not the case. The addition of HPC to the blend was intentional to stabilize the Polyox-poloxamer combination and to increase the versatility of the printing process. HPC, being a mucoadhesive polymer, provides a window to further use this formulation as a mucosal film formula with increased HPC concentrations. Poloxamer was added due to its solubilizing effect and its reported role in efficient printing with 3D printing techniques earlier [25]. The viscosity of the dispersions is a very important factor, as it determines whether they can be employed for the continuous 3D printing process, as considered for our research rationale. The polymers should be (i) thixotropic in nature, (ii) should increase their overall viscosity upon extrusion, (iii) should have appropriate viscosity for printing, and (iv)have sufficient mechanical strength after coming out of the printing nozzle [26]. The pseudoplastic rheograms for all the selected dispersions clearly indicate that these dispersions are suitable for extrusion. With an increase in the apparent concentration of the polymers, the apparent viscosity and storage modulus tend to increase for such pseudoplastic gels [27] which could be very crucial for shape retention and mechanical strength of the ODFs. But increased viscosity also could result in dimensional inaccuracy, which could fail the syringe extrusion process [28]. So, the maintenance of the dimensional accuracy of the filament/final structure and consistent shape fidelity factor closer to 1, without compromise in the viscosity, ensures that the final structure truly reflects the desired CAD model. Out of two technologies employed in this study, the syringe-pump based extrusion technique boasts better precision, as the former is a positive displacement-based method having better deposition control with retraction mechanism and repeatable start-stop mechanism [29]. The possible fluctuations in the pneumatic system could be avoided in the syringe system with its direct volume control, minimized dead volume, and reduced pulsation [30, 31]. Such precision is highly desirable if the final product involves pharmaceutical dosing or a complex architecture of regenerative medicine [32].

With dispersion-based semisolid extrusion, the primary limitation is that the CAD model is not achieved, especially for thickness. Lengthwise and breadthwise, there is not much change in the overall dimension during drying, but the thickness drop for the film is usually 60–75% based on our observation. It also depends on the concentration of polymers utilized in the solution. The thickness drops as the water present in the film evaporates and it is also depending on the drying time as well. The final formulation thickness for our batches were within the range of 100 −250 μm which is also a suitable thickness range for ODFs during patient handling [4]. The film could be further increased in size to increase more drug, if necessary, but thickness could be crucial in patient handling. Similarly, disintegration time is also a very important factor. There is no set specific limit or method for the disintegration time for ODFs. For ODTs, 180 s is listed in the European Pharmacopoeia, and ODFs also should have better disintegration due to their larger surface area [33]. For our formulations, the disintegration times range from 24 to 120 s, depending on the thickness of the film. Since the drug dose for SMT is slightly higher, we focused on designing the full five layers of ODF, which increased the thickness and overall disintegration time. For a low-dose drug, decreased infill could be a great possibility to improve the disintegration time. There are previous reports of thickness of 3D-printed and typical film thickness ranging within 100 μm or more [34] being acceptable if it retains acceptable mechanical properties and disintegration rate. In our earlier studies, we conducted many lower infill density films for their disintegration time, which had a very quick disintegration time portfolio for the same composition (data not shown). Infill modification indirectly alters the area of exposure to media/solvent and thus facilitates the disintegration/dissolution.

The surface morphology and roughness of the surface also hold an important place in regulating the release pattern, disintegration, and overall stability of the formulation excipients. The clarity of the surface, even after higher magnifications, ensures that the surface does not contain any defects or any sort of drug/excipient crystallization. The presence of any surface defects could be because of extreme dehydration during the drying step, and thus removing all the residual moisture in the films to completely remove their plastic nature [35]. The excessive drying, as observed after 60 min, as shown in Fig. 4, could be avoided to prepare better films. When infill is decreased, the drying time should be reduced proportionally as more surface area of evaporation dries it faster and the formation of such cracks is sooner. The surface roughness is optimal to drive the overall wettability of the surface, as evident with some of the previous ODF studies [28]. The surface roughness of the 3D-printed surface exhibited a clear drop in the contact angle with time, disintegrating the samples even within the contact angle experiment period. The lowering of the contact angle usually indicates better wettability, which could allow saliva to spread quickly across the ODF surface thus facilitating disintegration and dissolution. The presence of surface-active molecules in the oral films tends to adsorb at interfaces, reducing the interfacial Gibbs energy, thus lowering the surface tension. Sessile drop technique, either using static contact angle or volume change technique, gives a brief picture of how the dosage forms are wetted after coming in contact with liquid [36]. However, the viscosity of this liquid should also be given equal consideration.

Orodispersible films do not have reported guidelines for mechanical testing. However, a report by Preis *et al.* has shown the puncture strength of the marketed products [37] somewhere in between 0.08–0.4 N/mm^2^.Our fabricated ODFs of the lowest strength were found to be 0.72 N/mm^2^, which clearly shows that all the fabricated films are better in mechanical strength compared to the available ODFs. Mechanical strength is important, especially for the packaging cycles and the potable nature of the film. The stability of the drug in the film during storage is also essential. It was confirmed through our observation that the film, once prepared efficiently without structural defects and stored, retained its drug assay for 5 months. It was confirmed by thermal analysis data to ensure a solubilized or amorphous form of the drug in the film. The DSC studies also showed that the drug is converted into an amorphous form through 3D printing and through inhibition of crystallization by HPC; similar results were reported in the published literature [21]. Such crystallization inhibition property could be very crucial in formulation design of water-soluble drugs for orosdispersible films.

## Future Perspectives and Challenges

The utilization of innovative technologies like semisolid extrusion could be a great next step in the field of pharmaceutical manufacturing, as it brings in customization and personalization conveniently up to the patient. All the technologies utilized for ODF printing did not need any connections to the printer for fabrication, and one of the regular class syringes with the desired feed could be utilized for the fabrication. A similar approach was forwarded earlier about the use of a disposable syringe to obtain a 3D printer, which could be utilized for fast-dissolving delivery systems like ODFs [21]. The addition of polymers with mucoadhesive properties, like HPC, could help in translating this same product to a mucosal film with an increase in the concentration or through the utilization of higher viscosity grades. The films obtained from SSE were surprisingly strong in comparison to marketed films, as known from the published literature, which could be beneficial also in preparing multilayered mucosal patches for multiple drug release. The formulation nature as an ODF still keeps the absorption window considered after the stomach, but as disintegration happens in the mouth, the oral absorption is highly possible, and it could be translated through extension of this product to mucosal or sublingual films based on drug, dose, frequency window, and bioavailability. The concept of multilayered ODFs with multiple drugs could be pursued with a combination printing approach using two different SSE-based techniques together to incorporate multiple medications.

With multiple printing parameters involved in the fabrication process, there are lot of challenges to build a final structure with the desired consistency and precise loading. But with adversity, there is always a possibility to learn and grow. Pharmaceutical incorporation of 3D printing in large-scale production might require a few more years, as regulatory pathways are paving the way to streamline the overall pharmaceutical evolution into a translatable technology for regular drug product manufacturing. The extrusion-based system is still an inherently built as batch-based system rather than continuous, with higher costs of technology transfer and infrastructure setup. The usability of the reusable cartridges without affecting formulation stability should be ascertained as well [38]. Only selective formulations could achieve the shape fidelity as desired for extrusion-based manufacturing due to rheological limitations, thus also limiting its direct scalability into large-scale manufacturing [39].

A critical regulatory bottleneck 3D Printing manufacturing is facing for the past decades are- lack of standardized GMP frameworks based on manufacturing technique, sterility assurance guidelines, material traceability, and process validation [40]. The desired shift from industrial-scale manufacturing to point-of-care manufacturing has to be normalized by regulatory agencies with harmonized guidelines, specialized clinical audit operations for clinical manufacturing, and potential in-line risk assessment and process control [41]. The overall shift has been coming through this past decade, but it will definitely require more time However, IND approvals of a few tablet products produced using semi-solid extrusion-based technology (MED) in the past three years (T19,T20, T21 by Triastek, China) have given a huge hope for the next important milestone for 3D-printing and bringing the personalization out from the research lab to manufacturing plants [42].

## Conclusion

This work demonstrated the feasibility of the application of specific combinations of polymers using semi-solid extrusion to achieve faster disintegration. This is a starting point to prepare custom modified 3D-printed formulations, such as ODF, either to control disintegration, dissolution, or multiple drug delivery. The optimized batches were able to disintegrate within two minutes after contact with water, demonstrated content uniformity within 90–110% and showed impressive mechanical properties. The combination of polymers utilized was successful in preparing quick batches of faster-disintegrating ODFs. The addition of some functional excipients was crucial as they also helped in maintaining the film integrity, strength, and stability. No surface crystallinity was evident through surface morphology and through thermal analysis study for dispersion-based ODFs. Drying time showed crucial control over the formation of structural defects in the film, and regulation of both the in-process and post-drying steps was deemed suitable. This proposed method also provides an option to control the drug amount being loaded into the film through a multilayered approach or a change in the dimension of the film based on the required dosage and the patient’s need, thus facilitating personalization. This study is a comparative approach to investigate the feasibility of the SSE-based extrusion for pharmaceutical manufacturing; however, scalability of the process for large scale manufacturing, assurance of clinical sterility and overall improvement in the efficacy of production is another challenging step to truly translate such technologies from bench to bedside.

## Supplementary Information

Below is the link to the electronic supplementary material.

Supplementary file1 (DOCX 621 KB)

## Data Availability

We have the raw data available in lab note books and laboratory computer.
