# Supplementary material for: Comparative Formulation and Physicochemical Evaluation of Orodispersible Films Fabricated via Pneumatic and Syringe-Based 3D Printing
Source: Pharm Res. 2025 Nov 26;42(12):2205–21. doi: 10.1007/s11095-025-03967-4 (PMC12819439; doi:10.1007/s11095-025-03967-4)
Supplement: Supplementary file 1 — Supplementary file1 (DOCX 621 KB) [file 11095_2025_3967_MOESM1_ESM.docx]

**Supplementary Information**

**Comparative Formulation and Physicochemical Evaluation of Orodispersible Films Fabricated via Pneumatic and Syringe-Based 3D Printing**

Ishwor Poudel^1^, Nur Mita^1,2^, James Scherer^3^, Manjusha Annaji^1^, Xuejia Kang^1^, Oladiran Fasina^4^, Amit K. Tiwari^5^, R Jayachandra Babu^1*^

^1^Department of Drug Discovery and Development, Auburn University, Auburn AL 36830 USA

^2^Faculty of Pharmacy, Mulawarman University, Samarinda, Kalimantan Timur 75119, Indonesia

^3^Department of Biochemistry, Auburn University, Auburn AL 36830 USA

^4^Department of Biosystems Engineering, Auburn University, Auburn AL 36830 USA

^5^College of Pharmacy, University of Arkansas for Medical Sciences, Little Rock, AR 72205 USA

*Correspondance :

R Jayachandra Babu, Ph.D.

Email: ramapjb@auburn.edu

Phone: +1 334 844 8320

**List of Tables**

Table S1. Polymers screened for 3D-printing of ODFs using SSE

Table S2. Prescreening of various polymers and polymer combinations for feasibility with semisolid extrusion using pneumatic printhead.

Table S3. Mechanical characterization of ODF formulations using puncture test deformation of ODFs (using TA-52, 2 mm cylinder probe, force normalized to 100 μm thickness)

Table S1. Polymers screened for 3D-printing of ODFs using SSE

| Polymers | Product | Differential specifications |
| --- | --- | --- |
| HPMC | Methocel E3 | 3 mPa·s (2% solution in water at 20°C), Rapid hydration, faster dispersion, best suited in orodispersible films |
|  | Methocel E5 | 5 mPa·s (2% solution in water at 20°C), Rapid hydration, faster dispersion, best suited in orodispersible films |
|  | Methocel E15 | 15 mPa·s (2% solution in water at 20°C), Slower hydration, faster dispersion, best suited in orodispersible films |
|  | Methocel E4M | 4000 mPa·s (2% solution in water at 20°C). Provides strong film integrity |
| Polyplasdone | Crospovidone INF-10 | Rapidly swellable disintegrant, INF-10 refers to Type B, finer particle size and lower peroxide content. |
| Polyox | Polyox WSR 1103 | Viscosity is extremely high. Forms stronger films |
|  | Polyox WSR N10 | 30–50 mPa·s (5% aqueous solution at 25°C), Provides quick hydration in ODFs |
| HPC | HPC L | 6.0–10.0 mPa·s (2% aqueous solution at 20°C), Good film former |
|  | HPC LM | 11.0–20.0 mPa·s (2% aqueous solution at 20°C), Good film former, slower dissolution rate than HPC L |
| Maltodextrin | Maltrin M100 | Rapidly soluble up to 30%, contributes minimally to viscosity |
| Poloxamers | Pluronic F68 | Thermo-reversible gelation, highly water soluble. |
|  | Pluronic F127 | Thermo-reversible gelation, water soluble but more viscous and hydrophobic due to more polypropylene oxide content than F68 grade. |

HPMC: Hydroxypropyl methyl cellulose; HPC: Hydroxypropyl cellulose; ODFs: Orodispersible Films

Table S2. Prescreening of various polymers and polymers combination for feasibility with semissolid extrusion using pneumatic printhead.

| **Polymers** | **Transparency** | **Folding**  **endurance** | **Film**  **Brittleness** | **Disintegration** | **Printability** |
| --- | --- | --- | --- | --- | --- |
| Methocel E15(4% w/v) | ++ | ++ | - | <1 min | - |
| Methocel E4M (1.5% w/v) | + | ++ | - | <2 minutes | - |
| Pluronic F127 (2% w/v) | - | - | + | <2 minutes | +++ |
| E15(8% w/v)+ Polyplasdone (1.5% w/v) | - | - | + | <1 minute | -- |
| E15(8% w/v)  +E4M (1% w/v) +Polyplasdone(1.5% w/v) | -- | - | +++ | 2-3 mins | ++ |
| E4M(1.25% w/v)+E5 (10% w/v) | + | ++ | + | 1-2 minutes | ++ |
| E4M (2% w/v)+Poloxamer 188 (2% w/v) | - | ++ | ++ | <1 minute | ++ |
| Polyox (6% w/v)+Poloxamer 188 (1%w/v) | + | +++ | - | <20 seconds | ++ |
| Polyox (6% w/v) | + | + | - | <20 seconds | + |
| Polyox (6% w/v)+HPC(0.75%w/v)+Poloxamer 188 (1% w/v) | - | ++ | - | <30 seconds | ++ |

+++easiest, ++easy, +fair, -difficult, --extremely difficult, some observation and comparison were made with just single layer printing.

Table S3. Mechanical characterization of ODF formulations using puncture test deformation of ODFs (using TA-52, 2 mm cylinder probe, force normalized to 100 μm thickness)

| S.Code | Dimension | Weight,mg | Thickness, μm | Puncture force(N)* | Deformation (mm) | Deformation time(s) |
| --- | --- | --- | --- | --- | --- | --- |
| P-F6-Z | 20*20*1 | 100.4 ± 2.3 | 190.5 ± 10.1 | 4.34 ± 1.1 | 15.8 ± 0.7 | 31.6 ± 3.8 |
| P-F7-Z | 20*20*1 | 111.3 ± 2.3 | 304.8 ± 12.6 | 2.25 ± 0.7 | 17.2 ± 0.4 | 8.6 ± 0.4 |
| P-F6-Z-L | 20*20*1 | 119 ± 4.8 | 241.3 ± 9.6 | 3.04 ± 0.8 | 6.18 ± 2.3 | 12.3 ± 2.1 |
| S-F6-Z | 20*20*1 | 66.5 ± 2.5 | 127 ± 6.9 | 4.5 ± 0.4 | 10.1 ± 0.2 | 20 ± 2.5 |
| S-F6-Z-L | 20*20*1 | 81.1 | 152.4 | 3.6 | 7.85 | 20 |

**List of Figures**

Figure S1. (A) The ODF fabrication pipeline using Semi-Solid Extrusion Technology which employs selection of technologies and selection of excipients, before attempting final optimized ODF batch preparation and viability of the process, (B) The relative weight increment with layer addition in optimized P-F2-Z batch, (C) The relative increase in disintegration time with layer increment in P-F2-Z batch, (D) The increase in disintegration time with weight and increased infill for the P-F2-Z batch

Figure S2. The normalized disintegration time difference between semisolid extrusion-based technologies. All the ODF films were fabricated under the same dimensions

Figure S3. The experimental setup for the mechanical evaluation: (A) Fully stretched ODF at the end of tensile strength study: the ODFs showed localized stretch marked by loss of tensile strength rather than complete breakage, (B) The ODF was below the puncturing probe and puncture force to break the film was evaluated.

Figure S4. Wettability comparison of ODF batches fabricated by two SSE technologies using static contact angle method

Figure S5. Dissolution comparison of different batches of 3D printed ODFs: (A)Pneumatic batches printed with 25% and 50% infill ratio, (B) Pneumatic batches printed with different drug loading (1x, 2x), (C) Batches printed with different SSE technique (pneumatic vs syringe-pump extrusion), and (D) Pneumatic batches with inclusion of ascorbic acid in the final composition

**
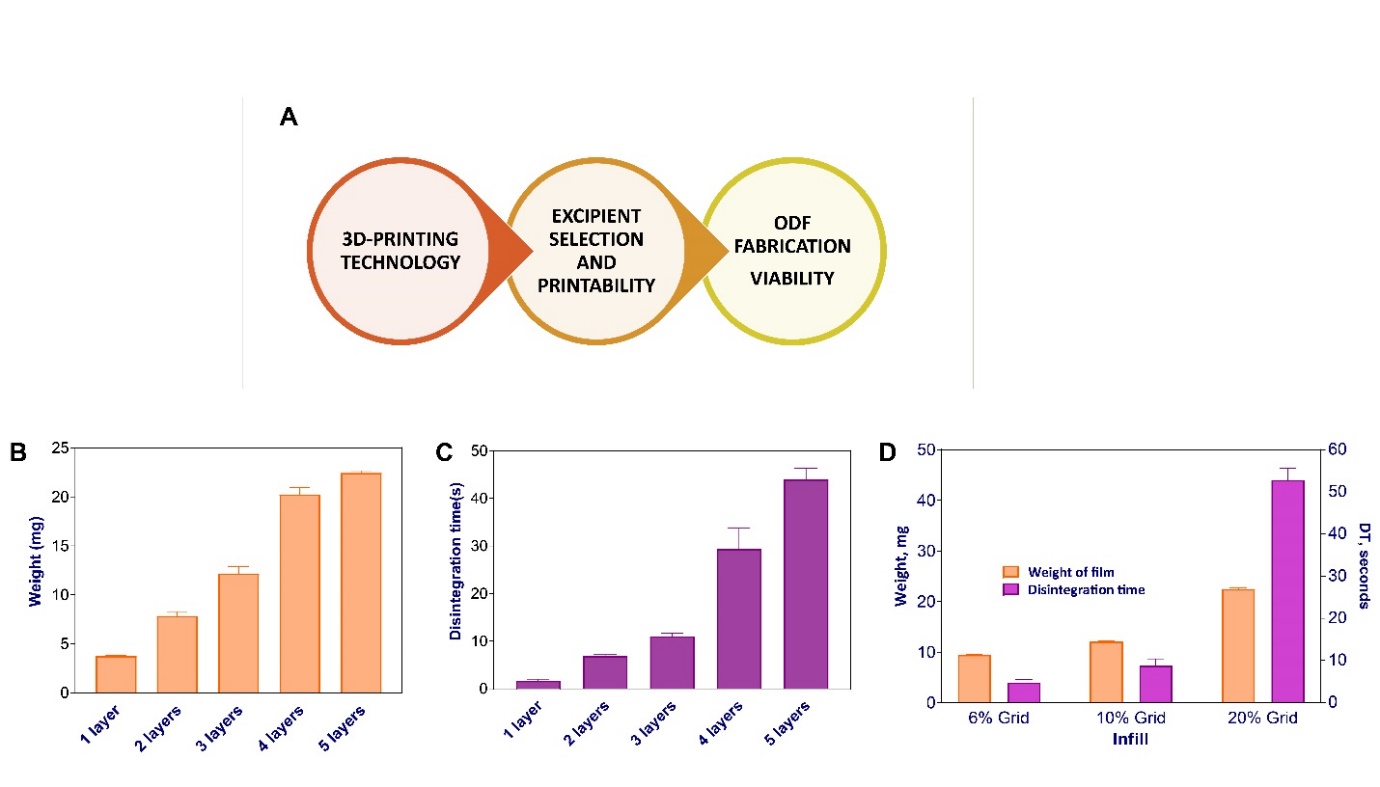
**

Figure S1. (A) The ODF fabrication pipeline using Semi-Solid Extrusion Technology which employs selection of technologies and selection of excipients, before attempting final optimized ODF batch preparation and viability of the process, (B) The relative weight increment with layer addition in optimized P-F2-Z batch, (C) The relative increase in disintegration time with layer increment in P-F2-Z batch, (D) The increase in disintegration time with weight and increased infill for the P-F2-Z batch


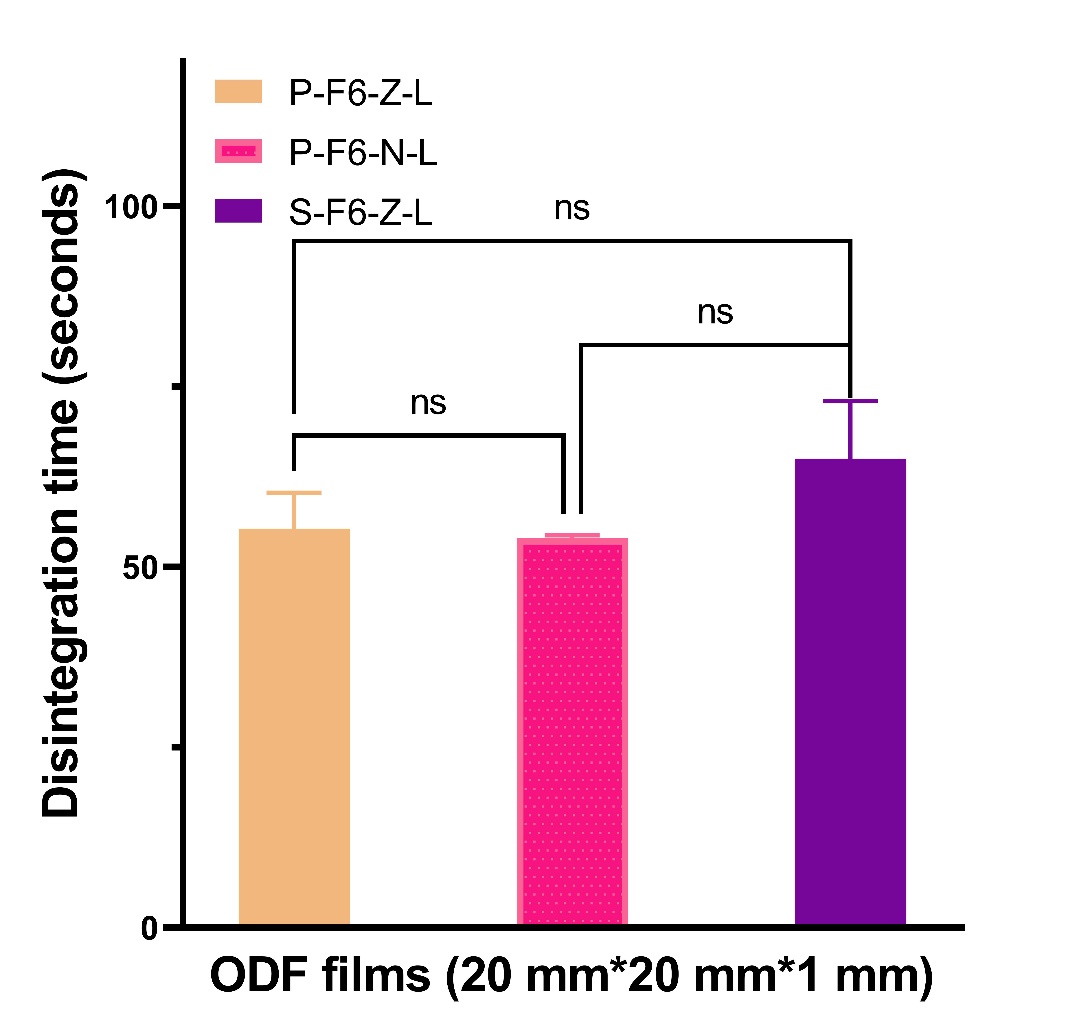


Figure S2. The normalized disintegration time difference between semisolid extrusion-based technologies. All the ODF films were fabricated under the same dimensions.


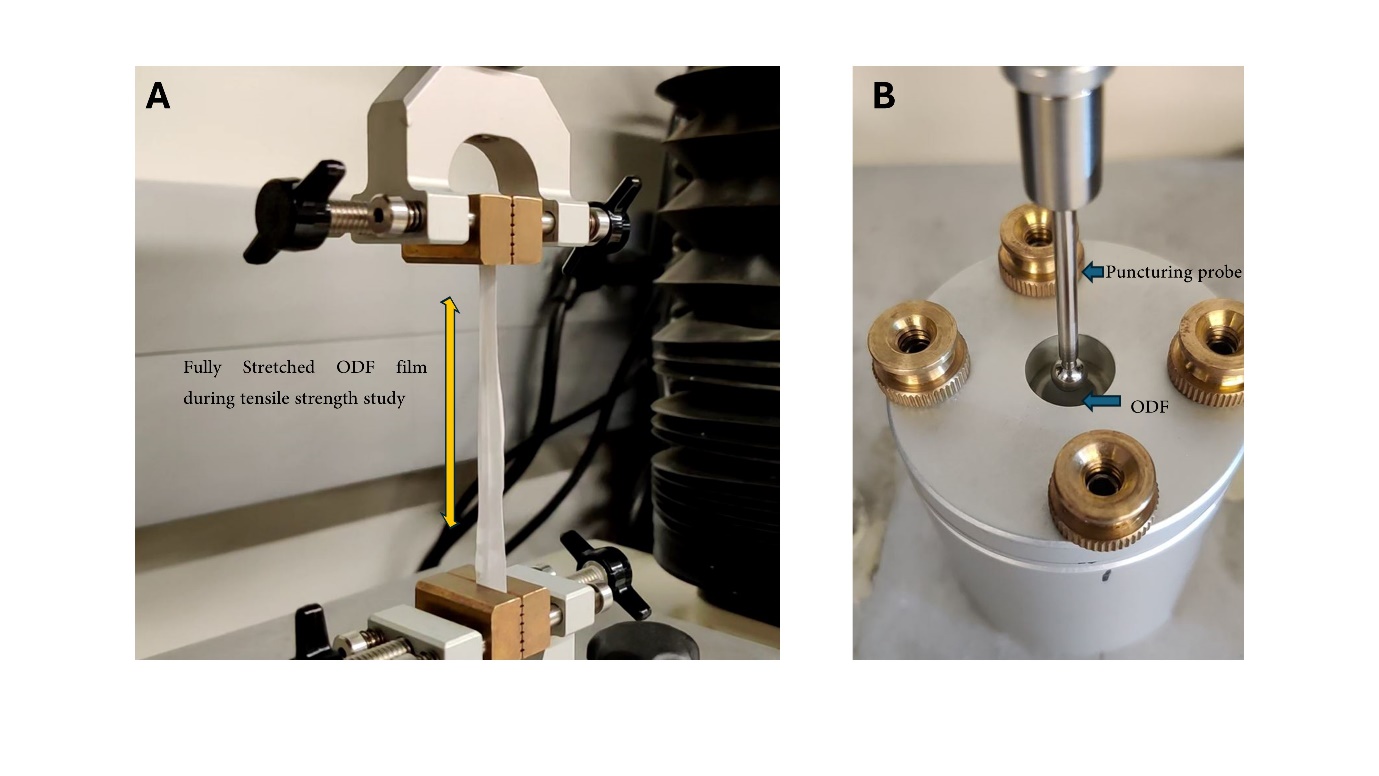


Figure S3. The experimental setup for the mechanical evaluation: (A) Fully stretched ODF at the end of tensile strength study: the ODFs showed localized stretch marked by loss of tensile strength rather than complete breakage, (B) The ODF was below the puncturing probe and puncture force to break the film was evaluated.


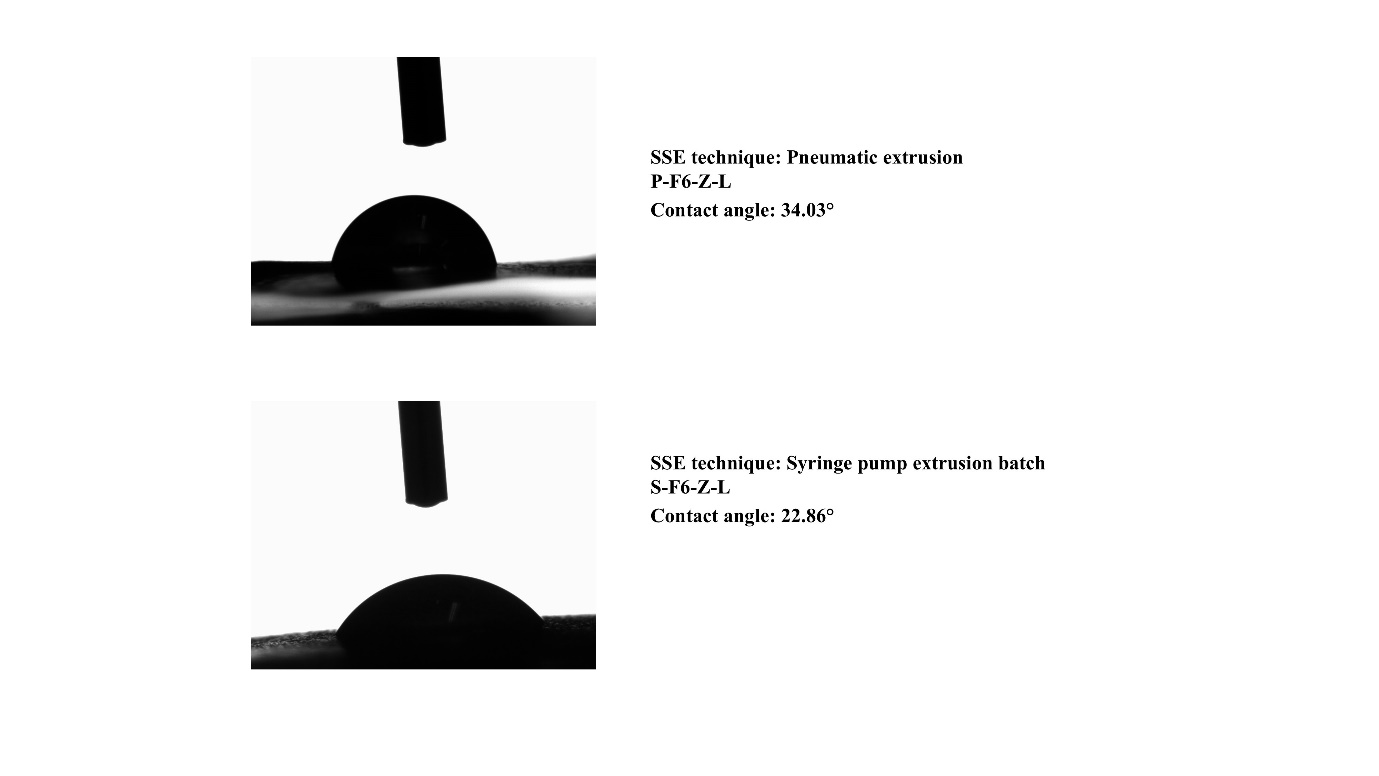


Figure S4. Wettability comparison of ODF batches fabricated by two SSE technologies using the static contact angle method.


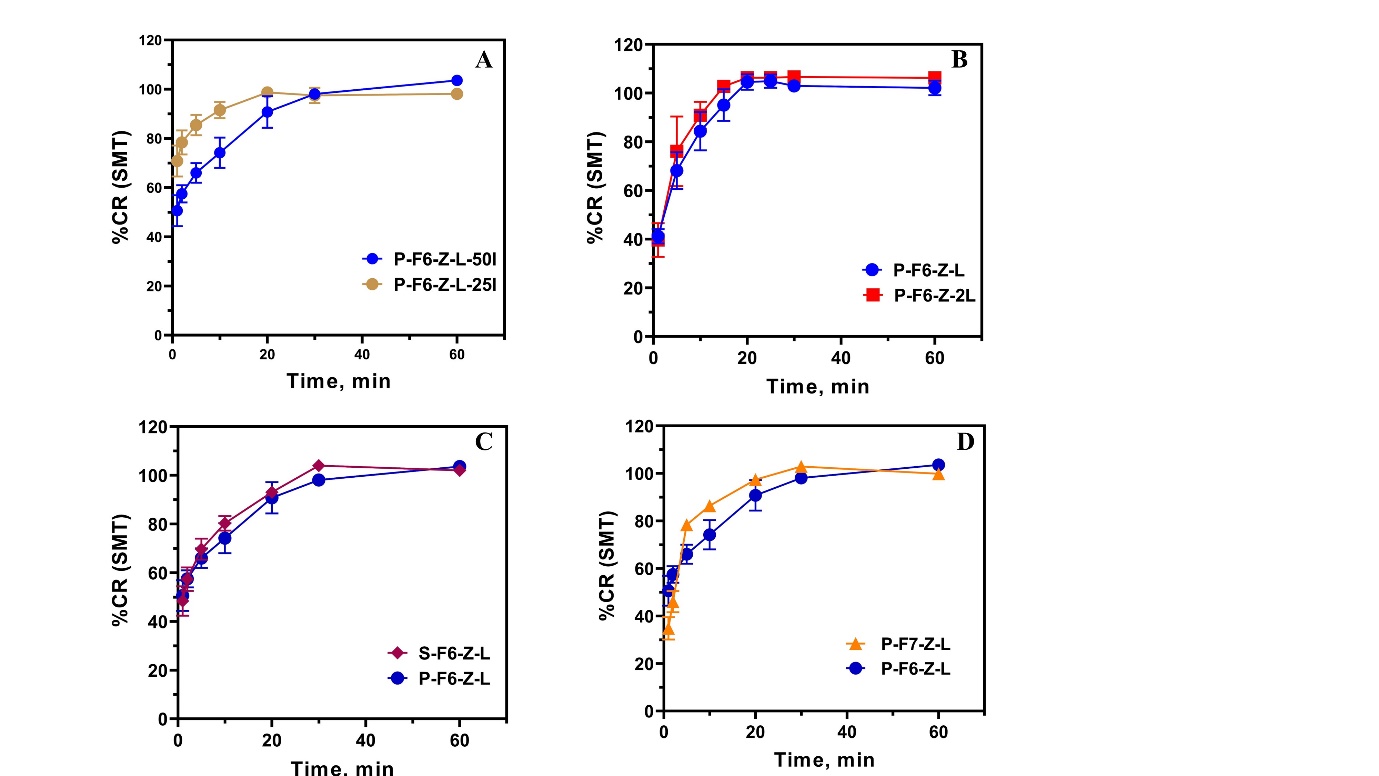


Figure S5. Dissolution comparison of different batches of 3D printed ODFs: (A)Pneumatic batches printed with 25% and 50% infill ratio, (B) Pneumatic batches printed with different drug loading (1x, 2x), (C) Batches printed with different SSE technique (pneumatic vs syringe-pump extrusion), and (D) Pneumatic batches with inclusion of ascorbic acid in the final composition
